# Deep brain stimulation mechanisms in comorbid pain and emotional dysregulation from animal models

**DOI:** 10.1016/j.isci.2026.117569

**Published:** 2026-09-23

**Authors:** Tian-en Si, Hao Han, Ying-ying Zhang, Zhen Zhou, Zhi-xiao Li, Jing Cao, Wei dong Zang

**Affiliations:** 1 Department of Anatomy, Basic Medical College, Zhengzhou University, Zhengzhou 450001, China; 2 The First Affiliated Hospital of Zhengzhou University, Zhengzhou, Henan, China

**Keywords:** deep brain stimulation, pain, depresssion, circuits, molecular pathways

## Abstract

Deep brain stimulation (DBS) is a potent neuromodulatory intervention for chronic pain and comorbid affective disorders. This review synthesizes preclinical evidence detailing the neural circuits and molecular cascades engaged by DBS in models of pain and emotional dysregulation. We delineate how DBS-targeting regions like the prefrontal cortex (PFC), thalamus, and nucleus accumbens (NAc) recruit the prefrontal-midbrain-spinal axis to facilitate endogenous opioid release and modulate neurotransmission, thereby attenuating nociception. Concurrently, stimulating the ventromedial PFC (vmPFC) or lateral habenula (LHb) ameliorates affective deficits by restoring serotonergic (5-HT) and dopaminergic (DA) tone and stabilizing circuit dynamics. By mapping these convergent and divergent mechanisms, we highlight how DBS fine-tunes reward, anti-reward, and descending antinociceptive pathways. This mechanism-based framework outlines a translational roadmap for personalized neuromodulation, guiding symptom-specific target selection and the development of biomarker-driven, closed-loop DBS systems to concurrently treat pain and affective pathologies.

## Graphical abstract

**Figure:**
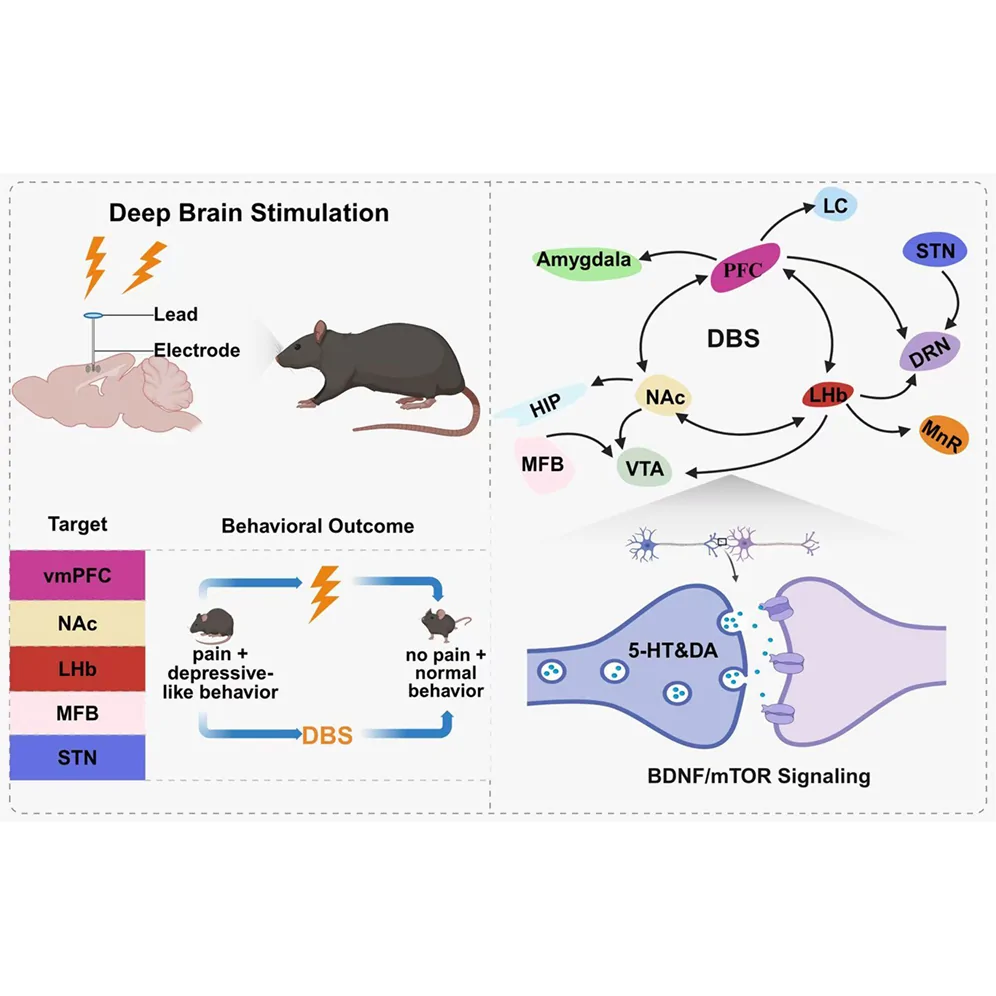

## Teaser

Neuroscience; Systems neuroscience

## Introduction

Chronic pain and comorbid emotional dysregulation (e.g., anxiety and depression) constitute a substantial global health burden, co-occurring to significantly impair patient quality of life.^1^^,^^2^^,^^3^^,^^4^ The pathophysiology of chronic pain extends beyond maladaptive hyperexcitability within the peripheral and central nervous systems, substantially altering cognitive, affective, and social functioning.^5^^,^^6^ Reciprocally, emotional dysregulation is underpinned by monoaminergic imbalances and maladaptive circuit remodeling, which collectively exacerbate nociceptive sensitivity.^7^^,^^8^ Although conventional therapies (e.g., pharmacotherapy, psychotherapy, and physical therapy) are effective in some patients; however, many remain refractory to these treatments, necessitating the development of more effective interventions.^9^

Deep brain stimulation (DBS), an adjustable neuromodulation technique, has emerged as a promising approach for treatment-resistant neuropsychiatric disorders.^10^ By delivering targeted electrical impulses to specific deep-brain nuclei, DBS modulates local neurochemistry and restores aberrant network dynamics.^9^ Unlike non-invasive modalities such as transcranial magnetic stimulation (TMS), DBS offers the unique advantage of precise, continuous modulation of deep-brain structures, rendering it a valuable therapeutic option for treatment-refractory cohorts.^11^^,^^12^^,^^13^ However, given its invasive nature and associated surgical risks, DBS is typically reserved for severe, chronic cases. Consequently, elucidating its precise neurobiological mechanisms is imperative for refining patient selection criteria and optimizing therapeutic parameters.

The pathogenesis of chronic pain involves aberrant activity across multiple brain regions, including the prefrontal cortex (PFC), periaqueductal gray (PAG), thalamus, nucleus accumbens (NAc), and insula.^14^^,^^15^^,^^16^^,^^17^ The PFC orchestrates the cognitive and affective dimensions of pain. In contrast, the PAG—a core hub of the endogenous antinociceptive network—governs critical descending pathways to the spinal cord.^18^^,^^19^ Additionally, the NAc, a key component of the reward system, mediates pain-related aversive experiences and emotional alterations in chronic pain.^20^ For instance, PFC-targeted DBS potentiates descending antinociceptive regulation and attenuates the emotional dysregulation of chronic pain.^21^ Thalamic DBS may reduce the persistence and intensity of chronic pain by modulating thalamocortical pathway activity.^22^ Studies indicate that DBS also improves pain-related neural circuit function by regulating glutamate, γ-aminobutyric acid (GABA), and noradrenergic signaling.^23^

Chronic pain and emotional dysregulation are closely interconnected, sharing interconnected neural substrates. The ventromedial PFC (vmPFC) is central to emotional processing; its dysregulated connectivity with limbic structures (e.g., amygdala and hippocampus [HIP]) sustains affective pathology.^24^^,^^25^^,^^26^^,^^27^ The NAc, a critical node in the reward system, regulates motivation and hedonic processing,^28^ while the lateral habenula (LHb)—termed as the brain’s “anti-reward center”—is pivotal in generating and sustaining negative emotions.^29^ DBS effectively ameliorates negative emotions by modulating activity in these regions.^9^ Studies demonstrate that vmPFC-DBS enhances prefrontal regulation of the dorsal raphe nucleus (DRN), increases 5-HT release, and upregulates 5-HT1B receptor expression, thereby alleviating anxiety and depression-like behaviors.^30^^,^^31^ Similarly, medial forebrain bundle (MFB)-DBS alleviates emotional dysregulation by augmenting dopaminergic (DA) transmission, reducing D2 receptor (D2R) expression, and enhancing reward system activity.^32^ LHb-DBS reduces anxiety and negative affect by modulating noradrenergic and DA signaling.^33^^,^^34^ Conversely, subthalamic nucleus (STN)-DBS may induce negative emotions by reducing 5-HT release, an effect potentially mediated by 5-HT1A receptors.^35^

The mechanisms underlying DBS-mediated rescue of pain and affective deficits rely on diverse neurochemical and molecular cascades. Specifically, 5-HT, DA, glutamate, and GABA serve as critical mediators in modulating both emotional states and pain perception.^36^^,^^37^ DBS alters synaptic plasticity and neural circuit activity by regulating the release of these neurotransmitters. For instance, DBS elevates 5-HT levels in the PFC and DRN, enhancing synaptic plasticity and ameliorating negative emotions.^31^ Furthermore, DBS drives the expression of brain-derived neurotrophic factor (BDNF) and modifies N-methyl-D-aspartate receptor (NMDAR) kinetics, thereby fostering long-term synaptic remodeling.^38^ In chronic pain models, DBS may suppress neuroinflammation, reduce pro-inflammatory cytokine levels (e.g., TNF-α and IL-1β), and attenuate neuronal hyperexcitability.^39^^,^^40^ Additionally, DBS modulates NAc and thalamic function to influence reward system activity, mitigating pain-associated negative emotions.^31^^,^^41^^,^^42^

Although previous reviews have extensively covered DBS applications in Parkinson’s disease (PD),^43^ obsessive–compulsive disorder,^44^ epilepsy,^45^ and isolated domains such as depression^46^ or chronic pain,^47^ an integrative synthesis addressing the comorbidity of chronic pain and emotional dysregulation is still lacking. Given their overlapping neural substrates and molecular underpinnings, this comorbid state not only presents profound therapeutic challenges but also serves as a critical window for decoding DBS mechanisms and optimizing neuromodulatory strategies. Therefore, this review aims to critically synthesize and contrast the target-specific mechanisms of DBS in preclinical models of comorbid pain and emotional dysregulation, highlighting the discrete neural circuits and molecular pathways engaged by electrical stimulation. By doing so, we provide a translational framework for advancing DBS from empirical open-loop paradigms toward precision-guided, closed-loop neuromodulation.

Our analysis underscores that modulating distinct targets (e.g., vmPFC, NAc, LHb, and STN) yields divergent—and occasionally opposing—outcomes. These target-specific effects stem from their differential engagement of discrete functional networks—namely, reward, anti-reward, and descending antinociceptive pathways—and their intersection with specific monoaminergic (5-HT and DA) and neurotrophic (BDNF) cascades. Decoding these target-specific mechanisms offers a translational roadmap for personalized target selection, tailored to whether a patient exhibits predominantly nociceptive or affective phenotypes. Future biomarker-guided interventions will ultimately hinge upon the seamless integration of patient-specific neuroimaging and multidimensional molecular profiling.

Beyond spatial targeting, therapeutic efficacy is heavily influenced by stimulation parameters. Mechanistic investigations elucidate how parametric variations (e.g., frequency) govern distinct neurobiological responses—such as the preferential enhancement of 5-HT release via high-frequency stimulation (HFS)—and drive long-term neuroplasticity through cascades like the BDNF/mammalian target of rapamycin (mTOR) pathway. Crucially, these insights are informing the development of next-generation bioelectronic strategies. First, adaptive DBS systems increasingly leverage real-time feedback from local field potentials (LFPs) or neurochemical fluxes to dynamically maintain optimal neural states. Furthermore, the advent of true closed-loop DBS relies on mapping the specific “failure modes” of underlying circuits. By utilizing discrete pathological signatures—such as aberrant burst firing in the LHb or uncoupled vmPFC-amygdala dynamics—as operational triggers, these advanced systems deliver corrective pulses solely upon detecting pathological deviations. This event-driven approach ensures highly precise, efficacious, and energy-efficient neuromodulation. Ultimately, deciphering the circuit and molecular underpinnings of DBS remains the fundamental driver for advancing neuromodulation into the era of personalized, closed-loop therapeutics.

## Search strategy and selection criteria

### Objective and review design

To synthesize preclinical evidence regarding the neural circuits and molecular pathways of DBS in animal models of pain and emotional dysregulation, we conducted was designed as a comprehensive narrative review. Rather than a quantitative meta-analysis, this review specifically emphasizes the mechanistic overlap and pathological intersection between chronic pain and negative affective states across different DBS parameters.

### Literature search strategy

A highly structured literature search was conducted in the PubMed database, primarily targeting literature published between January 2020 and May 2025 to capture the most recent significant advancements. However, foundational studies were also included without strict temporal constraints. The search queries utilized combinations of Medical Subject Headings (MeSH) and title/abstract keywords. Representative search strings included: (“deep brain stimulation” OR “DBS”) AND (“depression” OR “anxiety” OR “negative mood”) AND (“pain” OR “neural circuit” OR “molecular pathway”).

### Selection criteria and rationale for included targets

Literature was reviewed and selected based on its mechanistic relevance to the review’s core theme.

Inclusion criteria: we prioritized original preclinical animal studies that explicitly investigated DBS in the context of chronic pain, negative mood symptoms, or their comorbidity. Priority was given to studies offering detailed mechanistic insights (e.g., neuroplasticity, neurotransmitter dynamics, and neural circuit mapping).

Exclusion criteria: purely clinical efficacy trials, non-DBS neuromodulation techniques (e.g., TMS and spinal cord stimulation), neuroimaging-only studies, and DBS studies exclusively focused on motor symptoms in PD (without affective/pain assessments) were excluded to maintain a focused narrative.

Rationale for brain targets: the selection of specific DBS targets discussed in this review was driven by a clear anatomical and functional rationale. We selectively focused on structural nodes—such as the PFC, NAc, and specific midbrain/brainstem regions—because these areas serve as critical hubs where the ascending nociceptive pathways (pain perception) directly intersect with the cortico-striato-thalamo-cortical (CSTC) loops (reward and emotional regulation). Reviewing these specific targets allows for a precise elucidation of how single-target neuromodulation can simultaneously dictate both nociceptive and affective behavioral outcomes.

### Thematic synthesis

Relevant articles were imported into reference management software to remove duplicates. Rather than utilizing a strict double-blind screening protocol typical of systematic reviews, the literature was rigorously evaluated by the authors to extract key thematic elements. Data regarding the publication year, specific DBS targets, stimulation parameters, primary behavioral outcomes, and underlying molecular cascades were synthesized to construct a cohesive mechanistic narrative.

## vmPFC

As delineated previously, the vmPFC serves as a crucial integrative hub for cognitive, affective, and nociceptive processing; its dysfunction is a key contributor to the comorbidity of chronic pain and emotional dysregulation.^48^^,^^49^^,^^50^^,^^51^^,^^52^ Here, we synthesize preclinical evidence elucidating the circuit-level and neurochemical mechanisms through which vmPFC-targeted DBS ameliorates these comorbid pathologies.

### Anatomical features

Anatomically, the vmPFC occupies the ventromedial aspect of the PFC, extending into the medial orbitofrontal cortex. Rather than constituting a distinct anatomical entity, it functions as an integrated neural network comprising multiple Brodmann areas, including BA 25.^49^ In both humans and non-human primates, this region encompasses the medial orbitofrontal cortex and the subgenual anterior cingulate cortex (sgACC).^49^ In rodents, the primary functional homolog is the infralimbic (IL) cortex, which exhibits distinct cytoarchitectural and connectivity parallels to primate BA 25.^49^ Cytoarchitecturally, the vmPFC is composed of glutamatergic projection neurons and local GABAergic interneurons that form dense regulatory microcircuits. Furthermore, it maintains extensive, bidirectional connectivity with critical subcortical neuromodulatory centers, including the ventral tegmental area (VTA), locus coeruleus (LC), and raphe nuclei.^53^ Functionally, this prefrontal hub orchestrates executive control, social cognition, and affective processing. Crucially, it exerts top-down inhibitory control over the amygdala to extinguish aversive responses and attenuate maladaptive affective states.^53^

### Molecular mechanisms

#### Serotonergic system

The vmPFC exerts significant top-down control over ascending serotonergic pathways, a mechanism crucial for mitigating the affective comorbidities of chronic pain. In the chronic social defeat stress (CSDS) model, vmPFC-DBS effectively alleviates nociceptive and affective symptoms by increasing firing rates of 5-HT neurons in the DRN, thereby elevating extracellular 5-HT levels across multiple brain regions.^30^ The indispensability of 5-HT1B receptors in this process is highlighted by the observation that pharmacological blockade with the antagonist GR-1127935 significantly attenuates the anxiolytic and antidepressant-like rescue mediated by DBS.^30^ Moreover, in the chronic unpredictable stress model (CUS), HFS enhances DRN 5-HT neuronal activity, facilitating 5-HT release and improving affective states.^31^

#### Cholinergic system

Beyond monoamines, the cholinergic system serves as a critical effector for vmPFC-DBS. *Ex vivo* slice electrophysiology demonstrates that DBS significantly increases cholinergic tone, driving acetylcholine (ACh) release and altering cholinergic receptor dynamics.^54^ Muscarinic M1 receptors appear essential in this cascade; DBS-elicited after-depolarizations (ADPs) in vmPFC pyramidal neurons are critically dependent on M1-mediated transmission, which amplifies network excitability within the infralimbic cortex to facilitate affective restoration.^54^ In CSDS, acetylcholinesterase inhibitors (e.g., physostigmine) potentiate DBS-mediated antidepressant and anxiolytic effects, whereas cholinergic antagonists (e.g., scopolamine) attenuate its therapeutic efficacy. Mechanistically, cholinergic activation may improve synaptic plasticity and alleviate chronic pain and affective deficits by regulating astrocytic function and enhancing neurotrophic factor release (e.g., BDNF and nerve growth factor).^55^

#### BDNF/mTOR signaling pathway

At the molecular level, the therapeutic benefits of vmPFC-DBS are heavily reliant on neuroplasticity cascades, particularly the BDNF/TrkB and downstream mTOR signaling pathways.^56^ Notably, vmPFC-DBS markedly upregulates BDNF and mTOR protein expression, thereby rescuing stress-induced deficits in hippocampal synaptic density in rats subjected to chronic unpredictable mild stress (CUMS).^38^ Furthermore, DBS enhances ERK1/2 phosphorylation by activating the BDNF/TrkB pathway, thereby ameliorating depression-like behaviors.^57^ These findings suggest that the BDNF/mTOR signaling pathway may underlie the therapeutic efficacy of vmPFC-DBS for chronic pain and mood disturbances.

### Circuits

#### vmPFC-DRN

The vmPFC-DRN axis constitutes a pivotal pathway for the top-down modulation of affective valence and nociception. Synaptic architecture from the vmPFC exhibits distinct spatial topography within the DRN: projections preferentially innervate GABAergic interneurons in the lateral DRN, while concurrently modulating serotonergic output in the caudal domains. Under chronic stress, neural control from the vmPFC may lead to increased excitability of GABAergic neurons and decreased excitability of 5-HT neurons.^58^ Studies suggest that vmPFC-DBS enhances the discharge frequency of DRN neurons, thereby increasing 5-HT release, and exerting anxiolytic and antidepressant effects via a mechanism mediated by 5-HT1B receptors.^30^ The effect of DBS on improving DRN 5-HT neuron activity may be one of the key mechanisms through which vmPFC-DBS alleviates emotional disorders and pain.

#### vmPFC-LC

Beyond the serotonergic system, vmPFC-DBS recruits the noradrenergic LC to facilitate analgesia and affective stabilization. Specifically, DBS augments LC noradrenergic tone by upregulating tyrosine hydroxylase and desensitizing inhibitory α2-autoreceptors, effectively enhancing neuronal firing. Furthermore, DBS-induced neural oscillations optimize the functional coherence between the LC and the PFC, a process critical for sustaining antidepressant-like effects.^59^ Moreover, DBS modulates LC-cortical coherence, potentially optimizing functional connectivity and contributing to its antidepressant effects.^59^

#### vmPFC-amygdala

The amygdala serves as a nexus for aversive processing; its pathological hyper-responsivity frequently exacerbates the emotional burden of chronic pain.^60^ Current evidence indicates that the vmPFC provides essential inhibitory brakes on amygdalar output, a circuit that is characteristically compromised in comorbid chronic pain populations.^21^ It is therefore postulated that vmPFC-DBS restores this top-down inhibitory gating, thereby dampening amygdalar over-activation to suppress maladaptive emotional reactivity and nociceptive hypersensitivity.

In summary, vmPFC-DBS emerges as a multimodal intervention that transcends single-neurotransmitter models. By modulating multiple systems across the serotonergic, cholinergic, and noradrenergic systems—and fine-tuning signaling cascades like BDNF/mTOR—DBS modulates the altered connectivity between the vmPFC and subcortical nodes (DRN, LC, and amygdala) (Figure 1). While current findings underscore the potency of vmPFC-DBS in affective and nociceptive regulation, unraveling the precise cell-type-specific and temporal dynamics of these circuits remains paramount for translating these preclinical insights into individualized, precision neuromodulation therapies.

**Figure 1:**
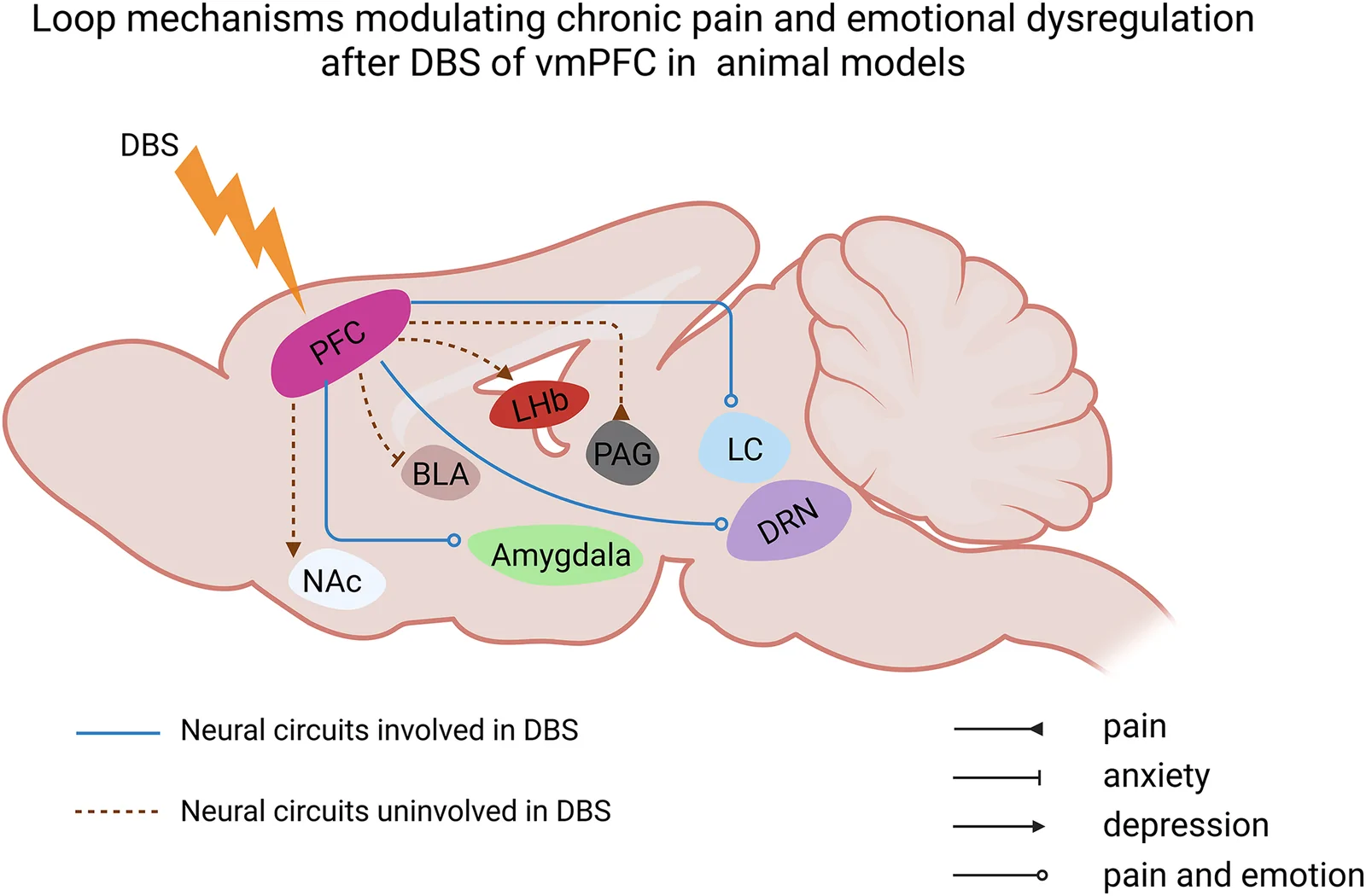
Neural circuitry mediating the therapeutic effects of vmPFC-DBS on chronic pain and comorbid emotional dysregulation in preclinical models The vmPFC mediates top-down modulation of nociception and associated aversive states via divergent descending projections to subcortical hubs (color-coded by anatomical targets). Solid lines denote empirically validated circuits directly engaged by DBS, whereas dashed lines represent pathways hypothesized to contribute to its therapeutic efficacy. Specifically, DBS recruits descending vmPFC efferents to the NAc, LC, and amygdala to drive the amelioration of pain and mood disturbances. Furthermore, these efferent targets dictate highly specific behavioral phenotypes: while vmPFC innervation of the PAG gates descending analgesia and vmPFC-NAc recruitment yields significant antidepressant-like outcomes, the aberrant activation of vmPFC-basolateral amygdala (BLA) or LHb axes characteristically provokes anxiogenic and pro-depressive responses, respectively.

## NAc

Positioned at the limbic-motor interface, the NAc serves as the brain’s central hub for reward and motivational processing. Dysregulation within this circuitry is a hallmark of affective pathologies, rendering the NAc a key target for neuromodulatory interventions in depression.

### Anatomical features

As the primary constituent of the ventral striatum, the NAc is anatomically parsed into a central core and a surrounding shell. While this dichotomy is well-conserved in rodents, the human NAc shell exhibits substantial cytoarchitectural complexity and cellular heterogeneity, further subdividing into medial and lateral subregions that govern functionally distinct aspects of reward and aversion.^61^^,^^62^ Cytoarchitecturally, the NAc is predominantly populated by GABAergic projection neurons, classically dichotomized into dopamine D1 receptor (D1R)- and D2R-expressing medium spiny neurons (MSNs).^63^ As a major integration center, the NAc receives dense, convergent glutamatergic afferents from the PFC (e.g., vmPFC and infralimbic cortex), amygdala, ventral hippocampus (vHPC), and thalamus, alongside dense DA innervation from the VTA.^64^ In turn, NAc MSNs send predominant inhibitory efferents to the ventral pallidum (VP), its primary downstream target, as well as to the substantia nigra pars reticulata (SNr). Together, these projections form the limbic-motor interface, completing essential CSTS feedback loops.^65^ Furthermore, in humans, the NAc acts as a highly evolved computational hub. Beyond processing basic affective inputs, it integrates complex cortico-striatal signals governing higher order cognitive phenomena, including outcome anticipation, cost-benefit decision-making, and self-referential processing.^64^ Consequently, the physiological operations of the NAc dictate reward valuation and action selection, while its maladaptive plasticity contributes significantly to neuropsychiatric conditions, notably addiction and major depressive disorder (MDD).^61^^,^^66^

### Molecular mechanisms

#### Neurotransmitter systems

NAc-DBS exerts significant antidepressant efficacy by modulating the dynamic interplay between monoaminergic and glutamatergic systems. Within the NAc, DA afferents classically modulate MSNs via D1Rs and D2Rs, mediating reward-seeking and aversive responses, respectively. Following CSDS, NAc-DBS upregulates both D1R and D2R expression and restores DA functional connectivity, thereby reversing stress-induced affective deficits.^62^ Concurrently, DBS substantially modulates glutamatergic transmission. By enhancing the synaptic recruitment and activation of α-amino-3-hydroxy-5-methyl-4-isoxazolepropionic acid (AMPA) receptors, NAc-DBS facilitates enhanced excitatory synaptic transmission, restoring critical limbic connectivity and suppressing depressive-like phenotypes.^62^

#### Neurotrophic factors

The upregulation of BDNF represents an important mechanism driving the neurorestorative and antidepressant properties of NAc-DBS. Chronic stress induces maladaptive structural plasticity and dendritic atrophy within limbic structures. NAc-DBS counteracts this by increasing BDNF expression, which promotes structural remodeling and mitigates stress-induced synaptic loss.^64^ Beyond local striatal effects, NAc-DBS promotes network-wide neuroplasticity. Notably, following CUMS, NAc-DBS effectively reverses the suppression of adult hippocampal neurogenesis (AHN) within the dentate gyrus (DG).^67^ Given that DG neurogenesis is indispensable for behavioral resilience, this long-range structural enhancement underscores BDNF-mediated cellular proliferation as a core therapeutic substrate of NAc-DBS.

#### Synaptic plasticity-related signaling pathways

At the intracellular level, the therapeutic efficacy of NAc-DBS is dependent on the activation of downstream cascade pathways, specifically the BDNF/TrkB axis. NAc-DBS triggers significant TrkB receptor phosphorylation, which promotes substantial synaptic structural remodeling and spine spinogenesis.^62^ Additionally, this cascade further recruits the mTOR signaling pathway. The activation of mTOR is necessary for local dendritic protein translation, a process required to sustain long-term potentiation (LTP) and rapid antidepressant responses.^64^ Furthermore, enhanced mTOR signaling is closely linked with metabolic homeostasis; by supporting the heightened bioenergetic demands of synaptic reorganization, NAc-DBS not only reinstates synaptic integrity but also fosters a resilient microenvironment against stress-induced cellular exhaustion.

### Circuits

#### NAc-PFC

The bidirectional functional connectivity between the mPFC and the NAc is essential for regulating decision-making and affective processing. Pathological states such as depression are characterized by a loss of top-down mPFC executive control over striatal hyperreactivity. NAc-DBS effectively restores this cortico-striatal coherence. Mechanistically, electrical stimulation in the NAc drives the antidromic activation of glutamatergic prefrontal afferents, thereby modulating mPFC pyramidal neuron firing. By restoring functional excitatory mPFC top-down inputs to the NAc, DBS re-establishes the delicate balance of the reward circuitry, enabling appropriate executive suppression of maladaptive affective states and promoting emotional resilience.^64^

#### NAc-HIP

The NAc-HIP axis links emotional processing with memory functions. NAc-DBS interacts with the hippocampal neural circuit, enhancing hippocampal neurogenesis and improving depressive symptoms. The neuroplasticity of the HIP is a key factor in emotional recovery. Studies show that in CUMS, NAc-DBS can enhance hippocampal neurogenesis and synaptic plasticity, reversing hippocampal maladaptive synaptic remodeling caused by chronic stress and improving depressive-like behaviors.^67^ Structural and functional remodeling of the NAc-hippocampal circuitry facilitates enhances in both affective regulation and cognitive function, providing a mechanistic basis for the multimodal therapeutic effects observed with NAc-DBS.

#### NAc-VTA

The NAc-VTA-DA pathway constitutes a core substrate for reward processing and emotional valence encoding. NAc-DBS potentiates VTA-DA activity through reciprocal NAc-VTA circuit modulation. Mechanistically, NAc-DBS disinhibits VTA dopamine neurons by suppressing local GABAergic interneurons, thereby enhancing mesolimbic dopamine release and reward responsiveness.^68^ Normalization of VTA-NAc circuit dynamics is essential for improving emotional dysfunction and alleviating depressive phenotypes.

Accumulating evidence suggests NAc-DBS as a multifaceted and promising neuromodulatory intervention for treatment-resistant depression (TRD). Rather than acting via a singular pathway, its antidepressant efficacy is driven by convergent pathways: from the modulation of monoaminergic and glutamatergic transmission, to BDNF/TrkB-dependent synaptic plasticity, and ultimately, the structural reorganization of large-scale networks. Specifically, the clinical outcomes of NAc-DBS rely on the restoration of functional integration across the NAc-VTA, NAc-PFC, and NAc-HIP circuits (Figure 2).

**Figure 2:**
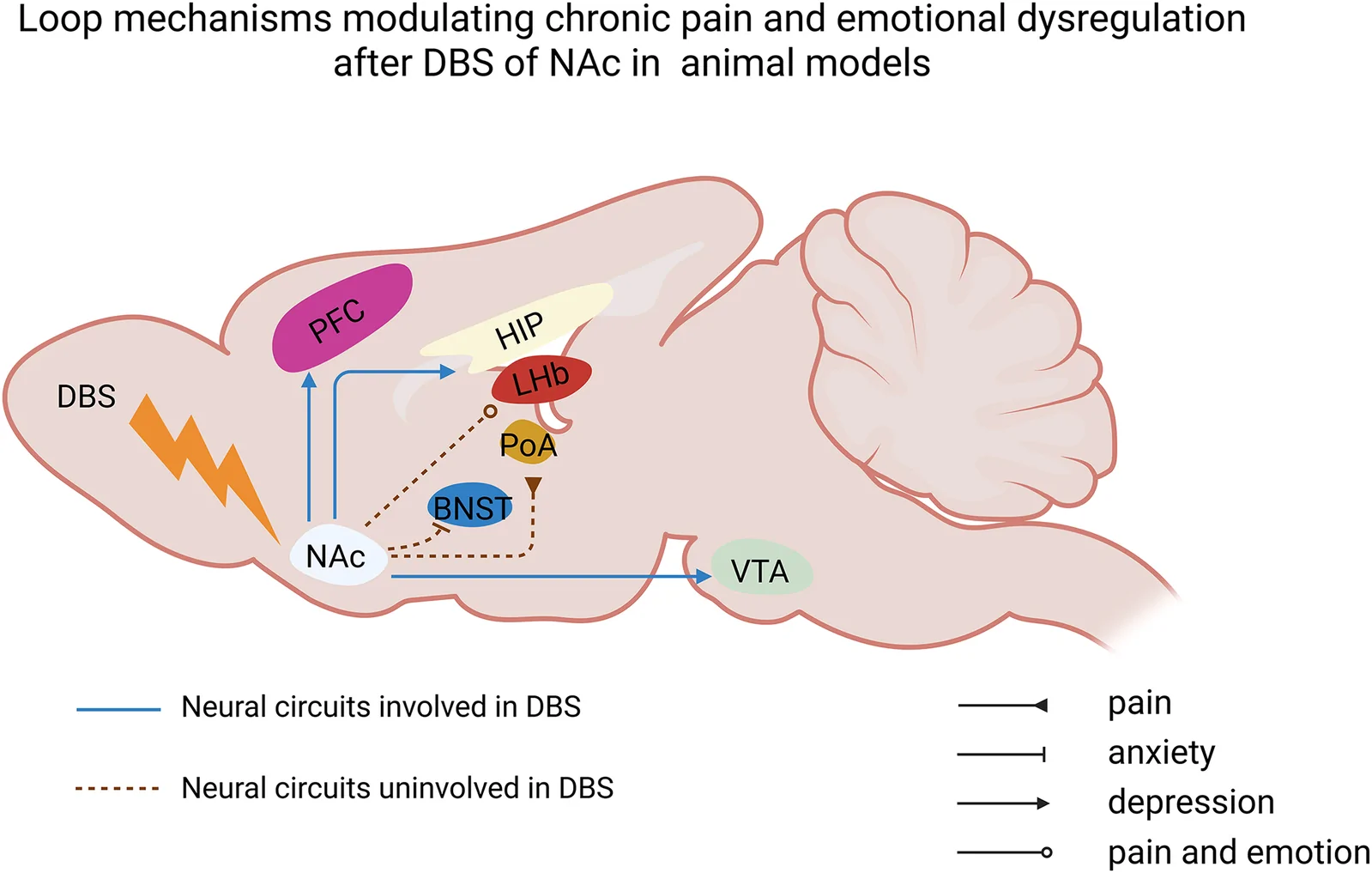
Neural circuitry mediating the therapeutic effects of NAc-DBS on chronic pain and comorbid emotional dysregulation in preclinical models After DBS of NAc, it projects to the downstream PFC, HIP, and VTA brain regions, thus relieving depressive symptoms. Whether DBS engages NAc projections to other downstream regions (e.g., the LHb or BNST) in modulating mood and pain remains unexplored. For example, NAc-lateral raphe nucleus relieves pain and mood disorders; NAc projections to the nucleus of the bed of the stria terminalis relieves anxiety, while projections to hypothalamic preoptic area (POA), a downstream brain region, have a role in pain relief.

To further elucidate the underlying mechanisms and optimize clinical implementation, future research should incorporate multimodal neuroimaging, electrophysiological mapping, and molecular profiling. While current efforts are primarily centered on depressive disorders, increasing insights into NAc circuit biology suggest translational relevance for chronic pain syndromes. Given the NAc’s dual involvement in affective processing and pain modulation, ongoing investigation may uncover novel mechanistic pathways through which NAc-DBS exerts analgesic effects, offering a potential avenue for innovative neuromodulation-based pain therapies.

## LHb

Recognized as the brain’s primary anti-reward center, the LHb exhibits marked pathological hyperactivity implicated in the pathophysiology of depression.^29^^,^^69^ Preclinical paradigms demonstrate that LHb-targeted DBS effectively attenuates this aberrant neuronal firing and restores monoaminergic tone, thereby significantly improving depressive-like phenotypes.^33^^,^^34^^,^^70^ This section delineates the anatomical, molecular, and circuit-level substrates underlying the therapeutic efficacy of LHb-DBS.

### Anatomical features

The habenula is a highly conserved epithalamic structure present across nearly all vertebrate brains. In mammals, it is positioned dorsomedial to the posterior thalamus and anatomically dichotomized into the medial habenula (MHb) and LHb. Substantial translational evidence highlights the LHb as a key node in the pathophysiology of MDD. Notably, across diverse animal models of stress and depression, the LHb stands out as a core region exhibiting prominent and persistent metabolic and electrophysiological hyperactivity.^29^^,^^71^ Functionally, the LHb serves as a critical convergence point—a macroscopic bridge linking forebrain cognition with midbrain neurochemistry. It receives dense projections from the limbic forebrain and basal ganglia, while sending dominant efferents to midbrain monoaminergic centers, particularly the VTA and DRN.^71^^,^^72^ This unique anatomical topology positions the LHb as a central hub for integrating value-based, sensory, and aversive experiential signals, thereby exerting significant top-down modulation over motivational, cognitive, and motor outputs.^73^

### Molecular mechanisms

#### Regulation of monoaminergic systems

LHb-DBS has shown observable antidepressant effects in animal models of depression. In CUMS rat models, LHb neurons exhibit pathological burst firing and hypersensitivity to aversive stimuli; however, LHb-DBS effectively reverses this abnormal firing pattern and decouples the synchronization between the LHb and VTA, thereby ameliorating depressive-like behaviors.^70^ Furthermore, DBS restores DA activity in the VTA, increasing DA release in the NAc, thereby improves depressive-like behaviors.^33^^,^^74^^,^^75^ Additionally, the serotonergic projection from the DRN to the LHb shows functional impairment in depression. LHb-DBS may alleviate depressive symptoms by enhancing serotonin (5-HT) transmission and suppressing excessive neuronal firing within the LHb.^34^^,^^76^

#### Regulation of calcium signaling pathways

In the learned helplessness model, LHb-DBS may exert antidepressant effects by regulating synaptic potentiation mediated by voltage-dependent L-type calcium channels, a process in which astrocytes may play a key role.^77^ In addition, LHb-DBS regulates the phosphorylation levels of calcium/calmodulin-dependent protein kinase II and glycogen synthase kinase 3, which are signaling pathways that contribute to the neuroplastic adjustments mediated by DBS and ultimately improve depressive symptoms.^39^

#### Regulation of energy metabolism and inflammatory signaling

LHb-DBS also impacts cellular energy metabolism and neuroinflammatory signaling pathways. Notably, LHb-DBS significantly downregulates the expression of CaMKIIα/β, GSK3α/β, and AMP-activated protein kinase in the orbitofrontal cortex in CUMS animals,^39^ suggesting that it may restore neural function through the modulation of energy metabolism pathways. Furthermore, LHb-DBS may influence the release of local immune mediators, attenuating neuroinflammation and providing an adjunctive mechanism for the treatment of depression.

### Circuits

#### LHb-VTA-NAc

The LHb projects to the VTA where it excites GABAergic interneurons that inhibit DA neurons, leading to decreased dopamine release in the NAc. This mechanism is believed to be a key factor in the anhedonia seen in depression. LHb-DBS may reduce the inhibitory influence of the LHb on the VTA, restoring normal DA neuronal firing patterns in the VTA and increasing dopamine levels in the NAc, thus alleviating depressive symptoms.^33^^,^^74^^,^^75^ Studies have shown that LHb-DBS reduces LFP power in the VTA and decreases the excessive response of VTA neurons to aversive stimuli, which further supports its antidepressant effects.^70^

#### LHb-DRN-5-HT

The LHb receives projections from the DRN and modulates its excitability via 5-HT receptors. Research has shown that a reduction in 5-HT transmission leads to abnormal firing in LHb neurons, which may mediate depressive-like behaviors.^34^^,^^76^ Optogenetic studies have demonstrated that activation of the DRN to LHb projection can reduce depressive-like behaviors in CUMS animals, suggesting this pathway may be a new target for depression treatment. LHb-DBS may alleviate depression by enhancing 5-HT release, reducing excessive LHb activity, and improving emotional dysfunction. Additionally, LHb-DBS may modulate the serotonergic transmission from the DRN to the LHb, affecting sleep, anxiety, and other associated symptoms.

#### LHb-median raphe nucleus

The LHb also projects to the median raphe nucleus (MnR), which plays an important role in regulating emotion and cognition. Studies have shown that the 5-HT system in the MnR is modulated by the LHb, and excessive LHb activity can reduce the excitability of MnR neurons, thereby affecting 5-HT release. LHb-DBS may restore MnR function, optimizing the balance of the 5-HT system and improving depressive-like behaviors.^78^ Furthermore, this modulation may involve stress response regulation, thereby mitigating stress-induced depressive phenotypes.

LHb-DBS improves depressive symptoms by regulating the monoaminergic system, calcium signaling pathways, and energy metabolism, optimizing the function of key neural circuits such as LHb-VTA-NAc, LHb-DRN-5-HT, and LHb-MnR. DBS restores DA and serotonergic activity in the VTA and DRN, reverses excessive LHb firing, and enhances neuroplasticity (Figure 3). Additionally, LHb-DBS may have a positive effect on stress responses, sleep, and other associated symptoms while modulating emotional behaviors. However, the precise mechanisms of LHb-DBS still require further investigation, and future studies combining optogenetics, electrophysiology, and molecular biology techniques will be crucial in exploring these mechanisms and optimizing its clinical application strategies.

**Figure 3:**
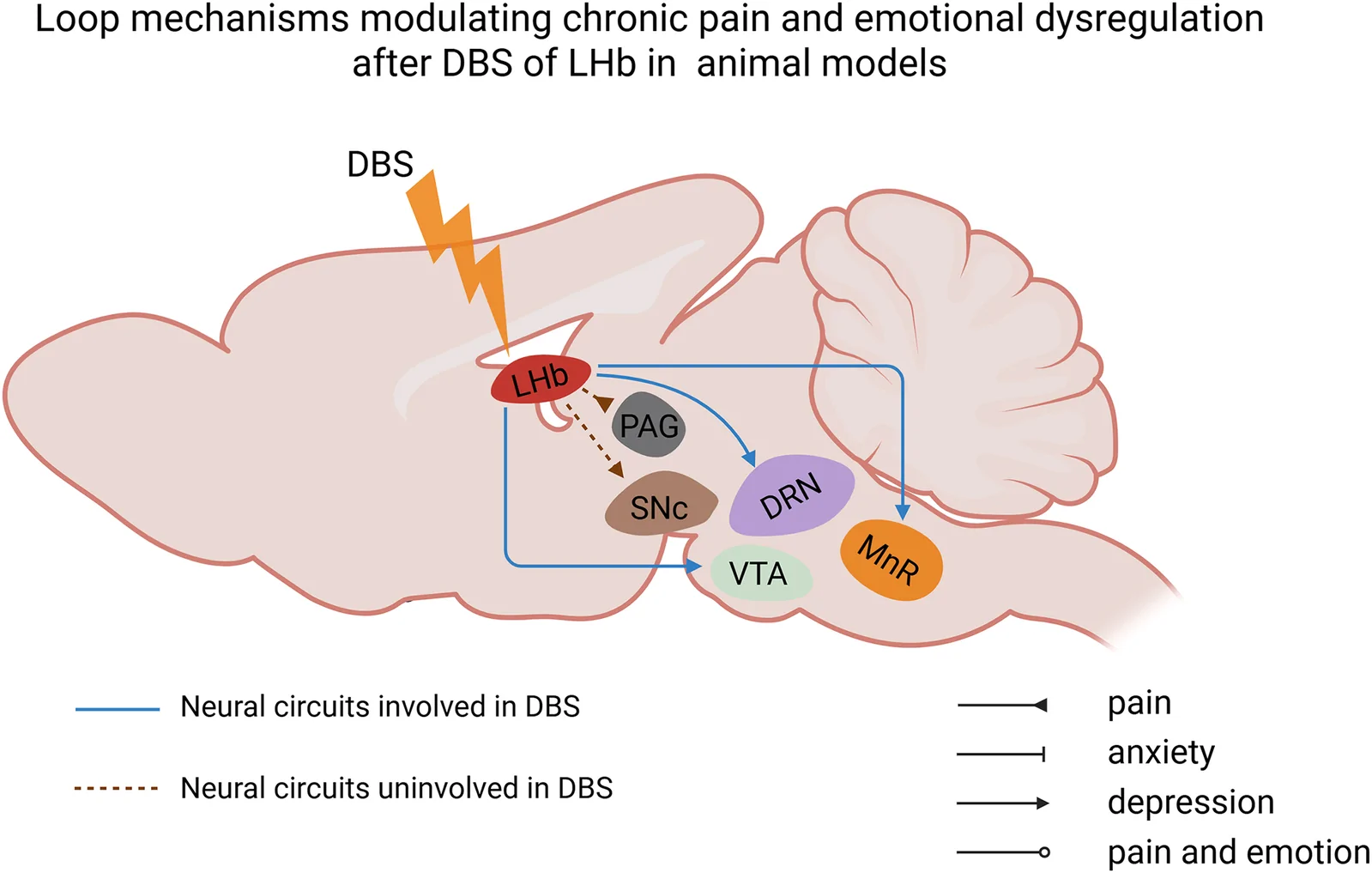
Neural circuitry mediating the therapeutic effects of LHb-DBS on chronic pain and comorbid emotional dysregulation in preclinical models The lateral habenula functions as the anti-reward center of the brain. Following DBS, the modulation of its efferent projections to downstream areas, including the DRN, MnR, and VTA, rescues monoaminergic tone and reduces depressive-like behaviors. Furthermore, the LHb projects to other downstream regions involved in emotional regulation, representing potential, yet-to-be-fully investigated circuit mechanisms for DBS efficacy. For example, future studies should explore the antidepressant effects mediated by the LHb-SNc pathway, or how projections to the PAG might relieve comorbid anxiety.

## MFB

The MFB is a major fiber tract connecting the VTA with the PFC and limbic regions and plays a central role in reward and motivation.^79^^,^^80^ As noted in the introduction, the MFB has gained attention as a promising DBS target for TRD. Here, we summarize preclinical evidence on the molecular and circuit mechanisms by which MFB-DBS exerts its antidepressant and potential analgesic effects.

### Anatomical features

The MFB is a complex white matter tract.^81^ From a neuroanatomical perspective, the MFB exhibits significant structural divergence between animal models and humans.^80^ In rodents, it has been classically described as a relatively discrete and unidirectional pathway projecting from the VTA to forebrain regions. In humans, however, advanced neuroimaging techniques have revealed a more complex trajectory, primarily integrated within the anterior limb of the internal capsule. Here, it is further subdivided into functionally complementary components: the superolateral branch of the MFB (slMFB), which mediates reward and motivation, and the anterior thalamic radiation (ATR), involved in processing negative emotions.^82^ This conceptual shift—from a “discrete pathway” to a “networked structure”—has deepened our understanding of the system and paved the way for the development of psychiatric neurosurgical interventions, such as DBS targeting the anterior limb of the internal capsule.

### Molecular mechanisms

#### Regulation of the serotonin system

Mechanistically, the therapeutic efficacy of MFB-DBS relies significantly on serotonergic modulation. MFB-DBS significantly upregulates the density of 5-HT fibers and increases 5-HT activity in the PFC and HIP, thereby regulating mood and cognitive functions.^83^ Furthermore, 5-HT1A receptors play a key role in the antidepressant effects of MFB-DBS. Notably, specific 5-HT1A receptor antagonists (e.g., WAY100635) can reverse the positive effects of DBS, underscoring the central role of the 5-HT system in the antidepressant mechanisms of MFB-DBS.^83^

#### Regulation of the dopamine system

MFB-DBS significantly modulates the DA system, particularly through projections from the VTA to the PFC and NAc. Acute HFS of the MFB increases the expression of the dopamine transporter (DAT) in the HIP and upregulates D2R expression in the PFC.^32^ Moreover, chronic MFB HFS in animals treated with clomipramine enhances the levels of DA, 5-HT, norepinephrine (NE), and their metabolites.^84^ These changes suggest that MFB-DBS can restore emotional reactivity by activating the DA system, thereby alleviating depressive symptoms.

#### Regulation of BDNF and neuroimmune systems

Neurotrophic signaling is another important component of MFB-DBS action. In CUMS models, MFB-DBS reverses the dysregulation of BDNF and neuroimmune mediators induced by chronic stress.^85^ By enhancing BDNF expression, DBS promotes restorative neuroplasticity in the PFC and HIP, which is essential for stabilizing mood, improving cognitive function, and ultimately mitigating depressive symptoms.

#### Modulation of the dopamine and NE systems in pain regulation

In addition to modulating emotions, MFB-DBS is involved in the regulation of pain perception. MFB-DBS can relieve chronic pain by modulating the release of DA and NE. Given their dual roles in mediating reward and emotional processing, these monoamines significantly influence the perception of pain. By stimulating the MFB, DBS can indirectly modulate the brain’s reward system, thereby reducing emotional responses to pain and altering pain perception thresholds.

MFB-DBS exerts significant antidepressant effects through the regulation of serotonin, dopamine, BDNF, and other molecular pathways. Furthermore, MFB-DBS demonstrates potential in chronic pain management by regulating the reward system and pain perception-related neural circuits. Although current research has revealed the role of MFB-DBS in negative emotions and pain relief, its exact mechanisms remain incompletely understood. Future work can integrate optogenetics, electrophysiology, and molecular biology to explore the mechanisms of MFB-DBS and optimize its clinical applications. As our understanding of the MFB’s role in pain regulation deepens, MFB-DBS is poised to become a novel therapeutic strategy for treating chronic pain.

## STN

The STN is a well-established DBS target for PD, but its stimulation can also modulate mood, sometimes leading to depressive side effects.^35^^,^^86^ This section reviews preclinical studies investigating the mechanisms by which STN-DBS affects emotional behavior, focusing primarily on serotonergic system alterations. Although STN-HFS has shown antidepressant effects in certain dopamine-related depression and anxiety models, these outcomes may partly be attributed to concomitant improvements in motor function. Functional imaging studies suggest that prior to STN-DBS, patients exhibit aberrant glucose metabolism in various brain regions implicated in emotional processing. Importantly, postoperative improvements in depressive symptoms are closely associated with the normalization of glucose metabolism in these networks.^87^ Additionally, chronic intermittent STN-HFS in the rat model reduces sucrose consumption and increases the immobility time in the forced swimming test (FST), indicative of depressive-like behaviors. Mechanistically, STN-HFS reduces the firing frequency of 5-HT neurons,^88^ decreases calcium activity in the cuneiform nucleus, reduces tryptophan hydroxylase-2 expression,^89^ and downregulates the mRNA levels of TrkB and BDNF in the HIP and amygdala.^90^ These findings suggest that the mood-altering effects of STN-DBS are mediated by disruptions in the serotonergic system, neuroplasticity, and energy metabolism. Clinically, this highlights the necessity for comprehensive psychiatric evaluations prior to STN-DBS in PD patients, as well as the optimization of stimulation parameters to mitigate these adverse effects.

## Discussion

The efficacy and behavioral outcomes of DBS in animal models are complex and highly target-dependent. For instance, although STN-DBS ameliorates motor symptoms, it can concurrently precipitate depressive-like behaviors by suppressing serotonergic neurons in the DRN.^89^ These findings underscore the critical need for precise parameter optimization to maximize therapeutic efficacy and minimize adverse affective and cognitive events. In the CUS model, vmPFC-DBS demonstrated significant efficacy, significantly reducing anxiety-like phenotypes and alleviating anhedonia.^31^ Stimulation of the LHb and NAc core yielded partial antidepressant-like effects, albeit less broadly efficacious than vmPFC stimulation. Sublenticular DBS (sDBS) directed at the infralimbic cortex gray matter induces ADPs in pyramidal neurons via cholinergic activation—an effect absent during white matter sDBS.^55^ Accordingly, extending stimulation pulse widths (e.g., from 90 to 400 μs) may optimally enhance cholinergic engagement in clinical applications^54^(Table S1). The antidepressant effects of infralimbic PFC (IL-PFC)-DBS depends on intact astrocyte function, which regulates neuronal excitability and network oscillations through adenosine A_1_ receptors and K^+^ buffering systems.^55^ Notably, glial dysfunction attenuated the efficacy of high-frequency (130 Hz), but not low-frequency (30 Hz) DBS, highlighting the potential for personalized frequency tuning to optimize outcomes.^55^ NAc-DBS may be more suitable for modulating reward and emotional circuits to improve anhedonia,^68^ whereas vmPFC-DBS may be more beneficial for enhancing cognitive function and synaptic remodeling.^38^ Despite the absence of direct NAc-to-PFC projections, NAc-DBS likely modulates prefrontal neurotransmitter release via the antidromic activation of corticofugal tracts, such as cortico-striatal fibers.^64^ DBS may also indirectly influence monoaminergic neurons in the midbrain and pons by modulating activity within the CSTC loop, thereby altering prefrontal neurotransmitter release.^64^ This DBS-induced enhancement of prefrontal monoamine release mirrors the neurochemical effects of combined selective serotonin reuptake inhibitor (SSRI) and antipsychotic therapies in refractory obsessive-compulsive disorders and MDDs, implying partially overlapping mechanisms. Because unmyelinated DA fibers exhibit higher electrical activation thresholds, the primary mechanism of DBS likely involves the recruitment of myelinated corticofugal fibers. Conversely, HFS of the SNr (SNr-HFS)—or STN-HFS acting via the SNr—modulates SNr-to-DRN projections. This suppresses DRN serotonergic neurons and blunts forebrain 5-HT release (e.g., in the mPFC), leading to anhedonia, avolition, and other depressive-like phenotypes.^35^ Patients with pre-existing deficits in the 5-HT system, such as those with PD, may be more susceptible to these affective adverse events. Furthermore, chronic STN-DBS (130 Hz, 80 μA, 60 μs) inhibits calcium signaling activity in DRN neurons. Via calcium-dependent transcriptional downregulation, this results in a phenotypic loss in serotonergic neurons (marked by attenuated TPH2 expression), ultimately blunting 5-HT synthesis and forebrain release.^89^ This mechanistic dissociation elucidates how STN-DBS can ameliorate motor symptoms while simultaneously inducing depressive-like behaviors (e.g., behavioral despair). Importantly, SSRI medications may prevent or reverse this DBS-induced 5-HT phenotype loss. Future research should further investigate how calcium signaling precisely regulates TPH2 expression and whether this process is reversible. Clinically, for patients with an existing risk of mood disorders, the globus pallidus externus (GPe) or internal globus pallidus (GPi) may be safer DBS targets. Additionally, further investigations are warranted to map the polysynaptic networks through which STN-DBS influences the raphe nuclei and the hippocampal BDNF system.

Preclinical evidence highlights substantial target specificity and mechanistic heterogeneity in DBS. Because regions such as the vmPFC, NAc, LHb, MFB, and STN are embedded within distinct functional circuits, their stimulation can elicit divergent—and occasionally opposing—behavioral phenotypes. For example, vmPFC-DBS alleviates negative affective states by enhancing prefrontal inhibitory control over the DRN, thereby increasing serotonergic neurotransmission in the forebrain.^30^ Conversely, NAc-DBS primarily reverses anhedonia by modulating VTA-DA tone, ultimately restoring the physiological dynamics of the reward circuitry.^67^ These mechanistic insights elucidate the variability in clinical responses across individuals and strongly support a “circuit-based” approach to target selection, moving beyond symptom-based criteria toward precision neuromodulation. Furthermore, converging evidence reveals shared neurobiological mechanisms underlying comorbid pain and mood disorders, including dysregulation of prefrontal-limbic connectivity (e.g., vmPFC-amygdala pathway),^21^ hypoactivity of the mesolimbic reward system (e.g., NAc),^62^ and hyperactivity of the LHb anti-reward center.^70^ The substantial overlap of these neural substrates provides a strong anatomical and functional rationale for single-target DBS interventions to achieve pleiotropic therapeutic effects. For example, vmPFC stimulation can concurrently recruit descending pain inhibitory pathways and limbic emotional networks, thereby enabling dual-domain symptom modulation.^21^ Beyond immediate circuit modulation, the sustained therapeutic efficacy of DBS relies significantly on substantial neuroadaptive changes. Preclinical studies indicate that efficacious DBS upregulates BDNF expression, activates intracellular growth-related signaling pathways such as mTOR, promotes hippocampal neurogenesis and synaptic plasticity, and modulates neuroimmune cross-talk.^38^^,^^67^ Collectively, these findings reframe the conceptualization of DBS: it functions not only as an electrical “neural pacemaker,” but as an active “initiator of neural remodeling.” Consequently, its long-term clinical benefits arise from the persistent functional and structural reorganization of widespread brain networks.

Beyond the canonical targets, several emerging neuroanatomical nodes have been identified. Notably, the bed nucleus of the stria terminalis (BNST) has emerged as a critical hub within emotion-regulating networks.^91^ Despite strong theoretical rationale, its clinical translation remains challenging. For instance, a recent double-blind crossover trial revealed that standard prespecified parameters failed to produce stable antidepressant effects, underscoring an urgent need for precise parameter optimization.^92^ To address this, circuit-specific modulation targeting the BNST-NAc projection has shown therapeutic promise in TRD. Furthermore, the efficacy of BNST-NAc stimulation correlates with dynamic shifts in low-frequency LFPs, particularly within the temporal dynamics of electroencephalogram (EEG) microstates.^93^ These findings suggest that the functional state of the BNST-NAc circuit may serve as a viable neurophysiological biomarker for predicting individual responsiveness to DBS intervention.^93^ Chronic DBS targeting the BNST-NAc region has been shown to produce significant amelioration of core depressive and anxiety symptoms. In contrast, acute or short-term stimulation selectively reduces dopamine D2R binding in key limbic and basal ganglia regions—including the amygdala, caudate nucleus, and substantia nigra.^94^ Furthermore, neurophysiological deficits—such as diminished BNST theta-band power and attenuated prefrontal-BNST coherence—are strongly associated with impaired top-down cognitive control, negative emotional bias, and trait anxiety.^95^ Collectively, these data highlight the BNST-NAc axis as a critical pathophysiological node and a highly translatable target for TRD. While this review primarily focuses on single-target interventions, clinical pain and mood disorders inherently arise from network-wide dysfunctions. This raises a critical frontier question: could the simultaneous or sequential stimulation of functionally complementary nodes—such as the reward-regulating NAc and the aversive-encoding LHb—yield combined therapeutic effects? Currently, preclinical evidence regarding multi-target DBS remain sparse. Theoretically, this strategy could more comprehensively remodel disrupted neural circuits. For example, combined stimulation of the vmPFC (which enhances cognitive control) and the NAc (which ameliorates anhedonia) may be more efficacious for patients presenting with both cognitive impairment and emotional apathy. Future research leveraging next-generation bidirectional bioelectronic interfaces is essential to map optimal multi-target combinations and spatiotemporal parameters in animal models. Such endeavors will ultimately pave the way for treating complex, refractory psychiatric and pain comorbidities.

By synthesizing the effects of DBS across diverse targets on pain modulation, affective processing, and neurotransmitter dynamics, this review underscores how mechanistic insights into neural circuitry can be directly translated into advanced clinical applications. Specifically, integrating multimodal neuroimaging—such as fMRI-based functional networks and diffusion tensor imaging (DTI)-derived structural connectomics—with neurophysiological and peripheral biomarkers will be pivotal for constructing reliable predictive models.^96^^,^^97^ Such models could guide personalized target selection—for example, vmPFC stimulation for patients predominantly exhibiting negative affect,^30^ and NAc/MFB stimulation for those with prominent anhedonia.^32^^,^^68^ Furthermore, translating preclinical parameter-response relationships (e.g., frequency and pulse width) into clinical settings provides a mechanistic framework for DBS programming. For instance, utilizing HFS to preferentially recruit the serotonergic system offers a rational alternative to heuristic trial-and-error titration.^31^ Finally, pathological neural signatures identified in animal models—such as aberrant burst firing in the LHb or characteristic oscillatory changes in LFPs within the PFC—represent key candidates for developing closed-loop DBS systems.^59^^,^^70^ A critical next step involves validating and standardizing these biomarkers in clinical cohorts to enable responsive, “on-demand” neuromodulation. By autonomously adjusting stimulation based on real-time, state-dependent biomarker fluctuations, closed-loop systems promise to maximize therapeutic efficacy, minimize off-target side effects, and optimize battery longevity. Ultimately, this dynamic intervention strategy holds significant potential to usher in a new era of precision bioelectronic medicine for complex neuropsychiatric comorbidities.

## Limitations and future directions for translation

Notwithstanding the considerable momentum in both clinical and preclinical DBS research, fundamental challenges and translational hurdles persist. A primary concern is the evolutionary divergence in neuroanatomy, functional circuitry, and affective processing, which inherently limits the translational validity of animal findings to clinical contexts. Furthermore, behavioral assays in rodents (e.g., forced swim or sucrose preference tests) provide only partial and indirect proxies for complex human affective constructs such as depression. Thus, interpreting these assays as direct phenotypic equivalents requires methodological caution. The lack of consensus on DBS parameterization—encompassing contact configuration, frequency, and pulse width—remains a major impediment to reproducibility and cross-study comparisons. Moreover, longitudinal safety and efficacy profiles for many target-specific protocols remain sparse. Finally, stimulation-induced neuroplastic changes may give rise to unintended neuropsychiatric sequelae (e.g., cognitive impairment or emotional lability), underscoring the need for systematic, longitudinal monitoring in clinical trials and practice. Addressing these challenges will require coordinated interdisciplinary efforts—spanning computational neuroscience, translational psychiatry, and clinical neurology—to develop more etiologically relevant animal models, implement cross-species validation frameworks, and establish prospective, standardized clinical registries to capture real-world outcomes.

## Conclusion

DBS has emerged as a promising neuromodulatory intervention for both TRD and chronic pain. DBS of different brain regions modulate affective pathology and pain relief by regulating specific neural circuits and molecular pathways.

Specifically, vmPFC-DBS engages the DRN pathway to modulate serotonergic projections and enhance synaptic plasticity. Conversely, NAc-DBS modulates DA tone, BDNF signaling, and neuro-metabolic homeostasis to restore reward circuitry, while exerting parallel modulatory effects on chronic pain. In the LHb, DBS effectively suppresses aberrant burst firing, thereby re-establishing the delicate equilibrium between the DA and serotonergic systems. MFB-DBS rapidly alleviates depressive-like behaviors through modulation of VTA-DA neuron excitability and widespread neurotransmitter regulation. Notably, while STN-DBS in PD can inadvertently induce depressive symptoms, optimally configured stimulation parameters can alternatively ameliorate such mood disturbances. Finally, PAG-DBS exhibits significant therapeutic efficacy for both nociceptive and emotional dysregulation, mediated predominantly via the endogenous opioid and monoaminergic networks (Figure 4).

**Figure 4:**
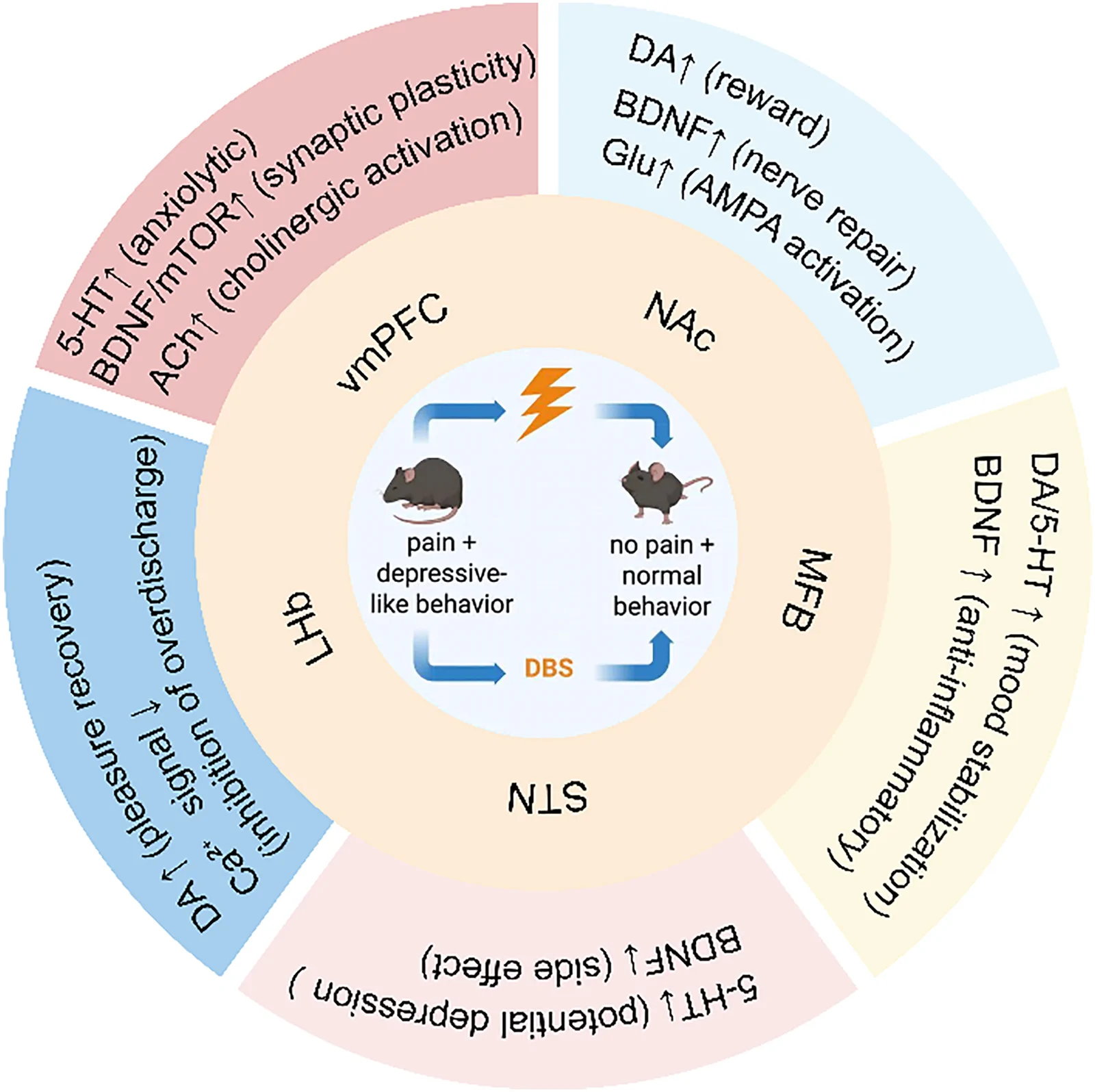
Molecular mechanisms underlying DBS effects on chronic pain and emotional dysregulation across different brain targets in animal models

Ultimately, the concurrent alleviation of pain and affective pathology by DBS relies on the modulation of spatially distinct yet functionally intertwined circuits—spanning vmPFC-raphe and NAc-VTA projections—alongside critical molecular hubs such as the 5-HT, DA, and BDNF/mTOR pathways. This granular, mechanism-based framework forms the theoretical foundation for next-generation neuromodulation. By tracking pathological electrophysiological biomarkers (e.g., region-specific oscillatory signatures) and leveraging individualized neuroimaging profiles, DBS can transition from empirical programming to precision medicine. Although the translational leap from animal models to clinical cohorts remains a formidable challenge, these deepening mechanistic insights are advancing DBS toward adaptive, personalized, and highly targeted bioelectronic therapeutics.

## Data and code availability

No datasets or code were generated or analyzed during the current study.

## Acknowledgments

This work was supported by the grants from the 10.13039/501100001809National Natural Science Foundation of China (grant no. 82371237). All figures are created with BioRender.com.

## Author contributions

Conceptualization, T.-e.S., H.H., J.C., and W.d.Z.; literature search and data curation, T.-e.S., H.H., Y.-y.Z., and Z.-x.L.; visualization, H.H. and Z.Z.; writing – original draft, T.-e.S. and H.H.; writing – review and editing, J.C. and W.d.Z.; supervision, J.C. and W.d.Z.; funding acquisition, J.C. and W.d.Z.

## Declaration of interests

The authors declare no competing interests.

## Declaration of generative AI and AI-assisted technologies in the writing process

During the preparation of this manuscript, the authors utilized ChatGPT (OpenAI, San Francisco, CA, USA) solely for language refinement and improving readability. Following the use of this tool, the authors thoroughly reviewed, verified, and edited the text to ensure scientific accuracy and integrity. The authors assume full responsibility for the final content of the manuscript.

## Supplemental information

Table S1. Activation of dentate gyrus PV + interneurons, restoration of gamma oscillations, and neurogenesis; involves NAc → VTA → DG pathway
